# A Narrative Review on the Application of Large Language Models to Support Cancer Care and Research

**DOI:** 10.1055/s-0044-1800726

**Published:** 2025-04-08

**Authors:** Ryzen Benson, Marianna Elia, Benjamin Hyams, Ji Hyun Chang, Julian C. Hong

**Affiliations:** 1 Department of Radiation Oncology, University of California, San Francisco, San Francisco, California; 2 Bakar Computational Health Sciences Institute, University of California, San Francisco, San Francisco, California; 3 School of Medicine, University of California, San Francisco, San Francisco, California; 4 Department of Radiation Oncology, Seoul National University Hospital, Seoul National University College of Medicine, Seoul, Korea; 5 UCSF UC Berkeley Joint Program in Computational Precision Health (CPH), San Francisco, CA

**Keywords:** large language models, clinical decision support, artificial intelligence, natural language processing, generative AI

## Abstract

**Objectives** : The emergence of large language models has resulted in a significant shift in informatics research and carries promise in clinical cancer care. Here we provide a narrative review of the recent use of large language models (LLMs) to support cancer care, prevention, and research.

**Methods** : We performed a search of the Scopus database for studies on the application of bidirectional encoder representations from transformers (BERT) and generative-pretrained transformer (GPT) LLMs in cancer care published between the start of 2021 and the end of 2023. We present salient and impactful papers related to each of these themes.

**Results** : Studies identified focused on aspects of clinical decision support (CDS), cancer education, and support for research activities. The use of LLMs for CDS primarily focused on aspects of treatment and screening planning, treatment response, and the management of adverse events. Studies using LLMs for cancer education typically focused on question-answering, assessing cancer myths and misconceptions, and text summarization and simplification. Finally, studies using LLMs to support research activities focused on scientific writing and idea generation, cohort identification and extraction, clinical data processing, and NLP-centric tasks.

**Conclusions** : The application of LLMs in cancer care has shown promise across a variety of diverse use cases. Future research should utilize quantitative metrics, qualitative insights, and user insights in the development and evaluation of LLM-based cancer care tools. The development of open-source LLMs for use in cancer care research and activities should also be a priority.

## 1. Introduction

Cancer remains the second leading cause of death worldwide, with nearly 600,000 deaths and approximately two million new diagnoses estimated in 2024 [ 1 ]. Cancer phenotypes and treatment strategies vary across patient subpopulations, clinical settings, and socioeconomic backgrounds [ 2 , 3 ]. Therefore, the role of data analysis and utilization in this domain holds critical importance. However, the complex and multifaceted nature of cancer is further complicated by the time and costs frequently associated with cancer care, and research data collection and analysis approaches [ 4 , 5 ].

A well-established body of literature has investigated the potential utility of natural language processing (NLP) within cancer research [ 6 7 8 ]. NLP has historically relied on rule-based, statistical ( *i.e.* , machine learning), and hybrid ( *i.e.* , rule-based and statistical) approaches to carry out various tasks on textual data. Over the past several years, NLP has undergone a major increase in profile. In 2018, Google released their seminal paper on bi-directional encoder representations from transformer (BERT) models, a large language model (LLM) based on transformer architectures from deep learning capable of incorporating bi-directional contextual relationships between words in text for NLP tasks [ 9 , 10 ]. These models gave rise to generative pretrained transformer (GPT) models, an encoder-decoder model architecture capable of taking input text and producing novel output text [ 11 ] . GPT models first gained wider notoriety in the scientific community following the release of GPT-3, an iteration of the GPT model that was shown to demonstrate state-of-the-art performance in various NLP tasks and capable of generating coherent and accurate human-like text [ 12 ]. Consequently, an increasing body of research is using LLMs to support aspects of cancer treatment and education.

This narrative review aims to describe advances over the past three years in the application and development of LLMs within cancer care, prevention, and education. We focus on two key NLP architectures with BERT- and GPT-based LLMs. Both have attracted significant attention: BERT is open source with widespread use and adoption, and GPT represents the current state of the art in NLP tasks. Furthermore, these model architectures are easily accessible for researchers, clinical providers, and organizational support staff, promoting equitable access to their use and accompanied by extensive support and documentation. This review summarizes key use cases, recent work, and future directions.

## 2. Methods

We performed a search of Scopus journal articles published in the English language, excluding reviews and articles published on preprint servers. We limited our review to studies published over the past 3 years ( *i.e.* , the start of 2021 through the end of 2023) to better reflect the contemporary state of LLM-based cancer research. Search keywords included ((“pretrained language model” OR “large language model” OR “llm”) OR (“generative pretrained transformer” OR gpt) OR (“bidirectional encoder representations from transformers” OR “BERT”)) AND ((oncology OR cancer)). Any additional articles of interest were retrospectively included. The resulting article titles and abstracts were screened for relevancy and kept where the primary focus was the use of LLMs within cancer care, education, and prevention activities. Four authors read the resulting articles while iteratively and inductively categorizing the articles into broad themes for qualitative analysis. Studies focusing on bioinformatics, genetics, and genomics captured in our study that were not directly related to cancer care were also excluded, along with studies in which the full text of the article could not be obtained.

## 3. Results

Our article selection approach yielded 51 peer-reviewed articles to be included in our analysis, along with an additional two studies of interest identified by the authors. LLM-based cancer research is in its infancy; therefore, cancer-focused LLM studies have demonstrated varying levels of study rigor and quality. When assessing the quality of LLM-based cancer research, readers should ensure that studies contain complete descriptions of the model, version, and available parameters used in the analysis, along with details on the model instance used ( *e.g.* , API-based vs UI/chat interface). Additionally, since LLMs are non-deterministic in the responses they generate, studies should assess the reproducibility of their findings by replicating experiments multiple times to ensure consistency in their output. Finally, higher-quality studies frequently incorporate domain experts in the experimental design and interpretation of model output. By incorporating clinical experts and end-users in the development and evaluation of these tools, studies can focus on ensuring that model-generated output is consistent with best clinical practices, accounts for unique and diverse clinical workflows, and minimize potential health and psychological risks posed to patients with cancer.

**Figure 1.**
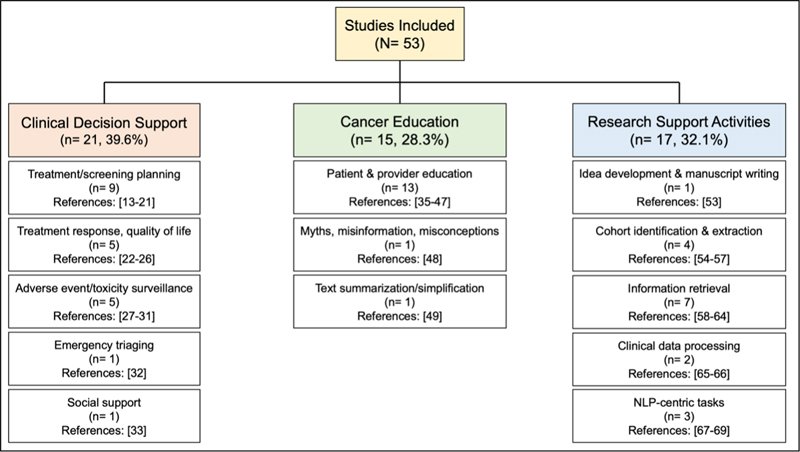
Broad themes and topics of articles in our narrative review.

The quality of studies included in our analysis varied and were often exploratory. Due to the lack of standardized LLM evaluation approaches in cancer research and clinical research more broadly, we observed varying levels of rigor in study design and model evaluation across studies. Studies often lacked critical details in their methods including whether API- or chat-based LLMs were used and which iterations/versions of LLMs were used. We focused our discussion on studies with well-described methods and results. Three primary themes emerged from the analysis: 1) clinical decision support, 2) patient and provider cancer education, and 3) support of cancer research activities. Figure 1 shows the distribution of primary themes and their foci across these studies.

## 4. Clinical Decision Support

The most frequently observed use of LLMs in cancer care focused on clinical decision support (CDS) activities. CDS can take shape in many forms but is typically characterized by providing clinical providers with information to assist in their clinical decision-making process. Selecting cancer treatment regimens that are effective and minimize adverse events is of paramount importance in cancer therapy. However, this process is nontrivial and highly dependent on numerous factors including patient cancer type, stage, genetics, and response to prior treatment.

### 4.1. Cancer Treatment and Screening Recommendations using LLMs

Research in cancer treatment planning has evaluated LLMs to assist with the recommendation of personalized cancer treatment regimens based on patient clinical histories, genetics, and current clinical guidelines [ 13 14 15 16 17 18 19 20 21 ], and has been largely limited to models using GPT architectures.

In a broad evaluation of cancer therapy recommendations, Bitterman and colleagues recently showed that when benchmarked against NCCN guidelines, GPT-3.5-turbo often provided incorrect treatment recommendations among other correct recommendations [ 14 ]. Lukac *et al.* , [ 13 ] observed similar deficiencies when they employed GPT-3.5 to make treatment recommendations for 10 breast cancer patients discussed at a multidisciplinary tumor board given various genetic components of the patient's cancer. They showed that GPT-3.5 demonstrated low coherence with tumor board recommendations for breast cancer therapies when provided with primary breast cancer cases in a standardized prompt format. Another study assessed breast cancer treatment regimen recommendations using LLMs, but augmented their prompting approach with relevant patient health data, and family history of cancer, along with patho- and immunopathological characteristics of the lesion [ 15 ]. Despite improved performance, the authors stated that their approach resulted in several instances of incorrect treatment regimen recommendations ( *i.e.* , hallucinations).

However, the application of GPT-4 to recommend treatment for fictional case vignettes of various cancers resulted in has shown to improve performance while also minimizing hallucinations as compared to GPT-3.5 [ 16 ]. This difference in performance is consistent with the findings of Rao *et al.* , [ 17 ] who used GPT-4 and GPT-3.5 to recommend breast imaging based on clinical presentation. They showed that when provided with a list of potential imaging modalities GPT-4 models performed consistently higher than GPT-3.5 models in making imaging recommendations for those presenting with breast pain and those undergoing screening. Similar performance was observed in a study by Lim *et al.* , [ 18 ] where they showed that GPT-4 models provided with colorectal cancer (CRC) and polyp management screening guidelines, performed significantly better in recognizing high-risk polyp features, recommending CRC screening intervals, and minimizing hallucinations compared to the base GPT-4 model.

### 4.2. Response to and Surveillance of Adverse Events Associated with Cancer Treatment

Of equal importance is the growing body of research focused on the application of LLMs to better understand responses to cancer treatment [ 22 23 24 25 26 ], mitigate adverse events and toxicities [ 27 28 29 30 31 ], improve triaging efforts [ 32 ], and provide social support for patients [ 33 ]. BERT models are more frequently used in this research avenue as they allow for further training of the model on specialized prediction and classification tasks with the addition of a neural classification layer on top of the original BERT model output [ 34 ].

Elbatarny *et al.* [ 22 ] conducted a study to classify metastatic disease response to treatments from radiology reports. The authors demonstrated that when grounding metastatic cancer response predictions using a standardized oncologic response lexicon (OR-RADS), BERT-based models outperformed human classification performance. Another study evaluated the performance of a fine-tuned BERT model (DFCI-ImagingBERT) in identifying cancer response and progression, using imaging reports of patients with non-small cell lung cancer [ 23 ]. They found that although their BERT model (DFCI-ImagingBERT) demonstrated superior performance to other NLP approaches, this difference was marginally better than computationally simpler methods including bag-of-words and convolutional neural network approaches.

In addition to evaluating disease response to treatment, the surveillance and management of adverse events and drug interactions during cancer treatment is important for triaging patients to increase attention as needed to mitigate acute care [ 27 , 28 ]. Studies have explored adverse drug reactions using both clinical and patient-generated health data sources, with variable performance. Zitu *et al.* [ 30 ] constructed a corpus correlating drugs with adverse drug events among patients who received immune checkpoint inhibitors. They then used ClinicalBERT, to detect adverse drug events at the sentence level from clinical records, which they did with high performance ( *i.e.* , F _1_ score: 0.87) [ 30 ]. A similar study using BERT-base to extract sentence-level adverse events from breast cancer patient-generated data [ 31 ] demonstrated lower performance ( *i.e.* , F _1_ score: 0.58) in identifying these events, demonstrating variability in the performance of BERT-based models for some clinical decision support tasks dependent on the use case, approach, and training data.

## 5. Cancer Education

At this point, an emerging use case of LLMs in cancer care has focused on medical education tasks. This natural fit for question-answer interactions has been exclusive to GPT models because of their ability to produce comprehensible and coherent information, as well as the ability to be further prompted for clarification, simplification, and summarization. GPT models have been used as an educational and question-answering tool for patients and radiation oncologists [ 35 36 37 38 39 40 41 42 43 44 45 46 47 ] for the detection and education of cancer myths, misconceptions, and misinformation [ 48 ], and to simplify and summarize radiology reports [ 49 ].

### 5.1. LLMs for Radiation Oncology Question Answering

We identified two studies using LLMs to answer questions related to radiation oncology to aid the oncologists themselves [ 35 , 36 ]. Holmes *et al.* demonstrated that when questioned on a set of 100 curated radiation oncology questions encompassing topics such as physics, brachytherapy, and imaging, GPT-4, on average, outperformed other LLMs and medical physicists. Huang *et al.* assessed the performance of GPT-4 in answering questions from the Radiation Oncologist in Training Exam (TXIT) [ 35 ]. They showed that GPT-4 was capable of achieving passing-level proficiency ( *i.e.* , >60%) on questions related to topics including but not limited to treatment planning, treatment toxicity, and disease prognosis. Despite admirable performance in answering various radiation oncology questions, both studies acknowledged that GPT-4 still struggled to correctly answer math-intensive questions, such as calculating radiation decay [ 35 ].

Aside from answering domain expert-focused questions, prior work has shown that GPT models are also capable of proficiently displaying the soft skills necessary for the safe and empathetic practice of medicine [ 50 ]. These capabilities naturally position GPT models as a promising approach for patient question-answering related to cancer care.

### 5.2. LLMs to Answer Questions of Patients with Cancer

Multiple studies have demonstrated that GPT-3.5 can accurately answer patient-related questions across a variety of cancers, but often struggles to present this information in a comprehensible way for non-expert audiences. Hermann *et al.* , [ 37 ] assessed the ability of GPT-3.5 to answer patient questions related to cervical cancer prevention, diagnosis, treatment, and survivorship. They showed that performance varied according to the types of questions being asked, generating more accurate results for questions on cervical cancer prevention, survivorship, and quality of life, and less accurate responses to questions of treatment. They also noted that responses felt as though they lacked compassion and personability. Musheyev *et al.* , used GPT-3.5 to answer the commonly googled questions about genitourinary malignancies including prostate, bladder, testicular, and kidney cancers. They showed that although answers to these questions were generally correct, they also found that responses lacked a suitable comprehension level for non-experts and lacked actionable information [ 38 ]. In another study, Johnson *et al.* , demonstrated near-perfect performance of GPT models for answering questions on common cancer myths and misconceptions but observed similar difficulties in the readability of GPT-answers by non-expert audiences [ 48 ].

These issues appear to be better mitigated in studies using GPT-4 for patient cancer education. Rogasch *et al.* , evaluated GPT-4 in answering questions about lung cancer and Hodgkin's lymphoma PET/CT reports [ 39 ]. They demonstrated that GPT-4 produced appropriate and meaningful answers while frequently displaying human-like empathy. However, when asked to provide answers to these questions with supporting references, GPT-4 investigators found that only 21% of answers contained valid and current references. Szczesniewski *et al.* , observed similar issues when prompting GPT-4 with patient-focused questions related to urologic malignancies. However, their prompting approaches did not explicitly ask for GPT-4 to provide supporting references to question answers [ 40 ]. Another study by Floyd *et al.* , found that GPT-4 performed similarly to GPT-3.5 in answering patient-centric radiation oncology questions in terms of accuracy and comprehensiveness, but outperformed GPT-3.5 when summarizing literature supporting current standard-of-care practice in clinical radiation oncology and in providing existing references to support claims [ 41 ].

These shortcomings in producing layperson language for cancer-related tasks further emphasize the need to better consider data augmentation and prompting methods proposed in computer science literature during study design. Some approaches that have been shown to often improve the performance of LLM in text generation tasks include few-shot learning which involves providing sample text as expected output to guide LLM generation [ 12 ]. Another approach is the use of retrieval augmented generation (RAG), which involves providing a model with access to an external knowledge source to guide text generation [ 51 ]. These external knowledge bases may include resources such as clinical treatment guidelines and published scientific literature, resulting in text responses better guided by evidence-based medicine. However, studies on the effect of RAG approaches to enhance clinical text generation by LLMs are still lacking [ 52 ]. Furthermore, more experimental work on various prompting strategies is needed within cancer research. Many studies in our review only reported on a singular standardized prompting approach or did not assess the reproducibility of LLM-produced output. In addition to technical limitations, much of the discrepancies in the lack of patient-readability and compassion of GPT-generated responses may be described by a lack of patient-centered design in the development and evaluation of these approaches. Therefore, further research focused on developing patient-facing applications for patient cancer education should utilize a user-centered design approach, incorporating the feedback of patients and members of the patient's support network, along with oncologists and domain experts in health communications.

## 6. Research Support Activities

Finally, we identified numerous studies focused on the use of LLMs in various research-related activities in cancer including research idea development and manuscript writing [ 53 ], cohort identification and extraction [ 54 55 56 57 ], information retrieval of drug information and clinical literature [ 58 59 60 61 62 63 64 ], clinical data processing for research [ 65 , 66 ], and various NLP-centric tasks ( *e.g.* , named entity recognition or NER, clinical note section segmentation) to support cancer research [ 67 68 69 ].

### 6.1. Assistance with Scholarly Research Activities

There has been much discussion throughout the scientific community about how best to ethically utilize generative LLMs in scientific research, including debates on their use in manuscript writing, grant preparation, and peer-review activities [ 70 , 71 ]. Aside from concerns regarding the lack of scientific rigor [ 72 ], much of this debate is centered around ‘hallucinations’ frequently occurring in LLM-generated text and the lack of some critical information in their responses. We only found one instance of assessing the use of LLMs for scientific writing within cancer. In this study, radiation oncologists with varying levels of research experience were asked to perform research tasks ( *e.g.* , literature review, hypothesis development, statistical analysis) with the assistance of GPT-3.5 [ 53 ]. Although inexperienced researchers found GPT-3.5 helpful for completing these tasks, the study also found that inexperienced researchers often believed in false GPT-generated information.

### 6.2. Information Retrieval and Extraction of Scientific Literature

Keeping current with scientific literature is becoming increasingly difficult and time-consuming due to the vast number of studies published each year. Aside from the sheer volume of papers, this process is time-consuming and requires comprehensive search strategies, abstract and full-text review, and interpretation of study relevancy. Zhang *et al.* , trained and employed a BERT model affixed with a text recurrent neural network (TextRNN) to identify cancer-focused literature and produce content-derived labels for each study. Their approach yielded very high performance, achieving an F _1_ -score of 0.93. However, at a more granular level, extracting meaningful individual elements from studies remains a difficult task. A study by Mutinda *et al.* , developed a BERT-based NER approach to automatically extract core elements of randomized controlled trials (RCT) including the study population, intervention, control, and outcomes from PubMed RCT abstracts [ 57 ]. Their approach demonstrated high performance when extracting these core elements, except for statistical analyses which the authors hypothesized was due to non-uniformity among included abstracts and the truncation of meaningful abstract information due to the BERT model's maximum input sequence length of 512 tokens.

These intricacies in cancer-related text are even more apparent in clinical text where typographical errors are incredibly common and can negatively impact the results of studies using this data. Lee *et al.* developed a BERT-based approach in which they used masked-language-modeling (a method in which the next word in a sentence is masked and predicted by the algorithm) in an attempt to identify and subsequently correct typographical issues present in clinical text [ 66 ]. They found that their approach yielded a higher performance in error correction compared to pre-existing methods, which is critical as information extraction studies remain very popular in clinical cancer NLP research.

### 6.3. General Information Extraction and Cancer Phenotyping.

Information extraction studies largely focused on extracting clinical features and phenotypes from clinical records for various cancers including breast, urologic, skin, and lung malignancies [ 55 , 56 , 62 , 63 ]. These types of studies are often a proof of concept, assessing the ability of NLP systems to create unique datasets for downstream tasks, whose findings are dependent on accurate extraction. LLM-facilitated information extraction not only enables robust data collection at scale but can also drastically reduce the time and costs typically associated with manual data extraction from clinical records [ 60 ]. The performance of LLM-based extraction systems, and most LLM-focused tasks in general, might be further enhanced through data augmentation and targeted prompt engineering approaches [ 12 , 51 , 52 , 73 ]. However, techniques in data augmentation and prompt engineering have still been understudied in the cancer literature and is an ongoing area of research.

## 7. Future Directions and Conclusions

Research and technological advances in LLMs continue to progress at a rapid pace, spurred by increasing computational resources and significant interest from the scientific community in their potential to enhance cancer care. Although this review focused on GPT- and BERT-based model architectures, some studies in our review compared GPT and BERT performances to currently available open-source LLMs such as GatorTron, Galactica, and Longformer [ 74 75 76 77 ]. However, the widespread development and use of open-source LLMs in cancer care and research is still lacking largely due to data privacy concerns, the immense computational requirements needed for their development, and ease of use for GPT- and BERT-based models by non-technical audiences. However, the LLM-developer community continues to innovate on open-source models, developing quantized LLMs ( *i.e.* , models with reduced computation and memory costs) that can be more easily deployed locally with less intensive hardware [ 78 , 79 ]. Additionally, open-source LLM models are readily available on public repositories such as the Hugging Face Hub, allowing for easier access to source code, documentation, and additional information on the models, to promote transparency in their function. Although proprietary closed-source LLMs such as the GPT series and the Llama models have demonstrated state-of-the-art performance in numerous NLP tasks [ 80 ], these models lack transparency in their training processes and model parameters, and trail the customization capabilities afforded by open-source models. However, the overall adoption of open-source models in cancer research is still lacking and future research should focus on developing and deploying open-sourced models to better protect patient data, increase the adoption of LLM technologies in diverse clinical settings, and increase trust in LLM-based technologies and clinical decision support provided by LLM-based applications.

In addition to increasing attention towards open-source models, there is a lack of standardized evaluation approaches for generative LLMs within cancer care and clinical care in general. Despite providing comprehensible responses and state-of-the-art performance for many clinical NLP tasks, LLMs remain limited in their capabilities, and human oversight of these technologies is essential [ 81 ]. Much of the computer science literature evaluates the performance of models against benchmark datasets such as the GLUE (diverse set of natural language understanding tasks) [ 82 ], MMLU (questions on 57 diverse subjects) [ 83 ], and SQuAD (100,000 + human-generated question-answer pairs) [ 84 ] datasets, to name a few. Such approaches are not as feasible in medicine due to privacy concerns with patient data, insufficient training data, and the inherently complex nature of clinical care [ 85 ]. As a result, there remains a critical gap in evaluation metrics for medical applications of LLMs, and both quantitative and qualitative evaluation approaches are needed, along with standardized principles for LLM-text generation in medicine [ 86 , 87 ]. Qualitative metrics are incredibly important in the evaluation of clinically focused LLM applications, and we encourage future research to incorporate the perspectives of patients, caregivers, healthcare providers, and domain experts during the development of these tools to ensure their optimal fit, adoption, and effectiveness among end users. Further, human oversight of LLM-generated clinical information remains critical to ensure completeness and accuracy while minimizing potential health and psychological risks associated with their use in cancer.

The heterogeneity in study design, use cases, and evaluation approaches of studies included in our analysis underscore the need to regulate the development, deployment, and use of large language models within cancer. Throughout the scientific community, there has been a stated need for the regulation of LLMs in healthcare to promote data privacy and governance, ensure patient safety, and improve clinical processes. [ 88 , 89 ] In the clinical setting, LLMs are becoming increasingly exposed to and or trained on patient-specific health information including patient-generated data ( *e.g.* , patient-authored messages in patient portals) and EHR data. Although these data sources are rich in clinical information, regulatory oversight of LLMs in cancer care can help ensure that patient data is not compromised or leaked through LLM-generated text and that clinical decision support and patient-facing applications using LLMs pose minimal risk to patients.

Finally, in addition to the need for regulation, rigorous model evaluation approaches, and more open-source LLMs, future research should look to bolster the reliability, clinical utility, interpretability, and accessibility of these models in cancer care. Current research opportunities include the need to improve the reliability and accuracy of LLM-generated output by training open-source LLMs on clinically meaningful text such as standardized ontologies, oncologic treatment guidelines, and deidentified EHR data, to name a few. Additionally, the interpretability and accessibility of LLM-generated content can be bolstered by increased development and deployment of open-source models in cancer research and by developing better approaches for visualizing LLM-generated output in clinically meaningful ways ( *e.g.* , grounding LLMs in knowledge graphs, user-centered design of LLM user interfaces, further establishing platforms for hosting and using LLMs).

Our study has several limitations to consider. First, when screening for articles, we queried Scopus and did not include additional scholarly databases such as MEDBASE or Google Scholar. This was deliberate as much of the research focusing on the application of LLMs in cancer is not Medline-indexed and is published in computer science or NLP proceedings. Additionally, our search strategy was limited to studies using BERT- and GPT-based LLMs. We limited our analysis to BERT models due to their sustained use over the past 4 years and extensive documentation, and GPT models due to their current status as the state-of-the-art LLMs for NLP-related tasks, and the widespread integration of GPT-instances into the EHR and EHR software.

Second, we restricted our final articles to journal articles only, excluding reviews and articles posted on preprint servers. Although informative, we excluded review articles to provide primary accounts of the constituent research advances in the application of LLMs to cancer. Additionally, we excluded preprint articles as they are not formally peer-reviewed and published. By excluding studies posted on preprint servers such as arXiv and medRxiv, our review may exclude applications of open-source models in cancer and salient findings outlined in these studies. Finally, cancer-related terms included in our search strategy were limited to “cancer” and “oncology”. As a result, studies containing specific cancer terms or disease names ( *e.g.* , “glioblastoma”) and not one of “oncology” or “cancer”, may not have been captured in our search strategy. Since the encompassing themes of our review were inductively identified based on our data collection, additional studies focused on LLMs for cancer care containing excluded keywords, may not have been captured by our search strategy.

In conclusion, the prospective impact of LLMs to support and enhance cancer care is highly apparent. These technologies hold the potential to transform clinical care by reducing burdensome clinical workloads, improving patient and caregiver understanding of cancer, and supporting scientific discovery and clinical advances. However, now more foundational work on the capabilities, limitations, and evaluation approaches of these technologies is needed prior to implementation within clinical care, along with greater utilization of open-source LLMs to promote model transparency, protect patient data privacy, and improve the interpretability of clinical decision support applications using LLMs.
